# Nicotinamide Ameliorates Dextran Sulfate Sodium-Induced Chronic Colitis in Mice through Its Anti-Inflammatory Properties and Modulates the Gut Microbiota

**DOI:** 10.1155/2021/5084713

**Published:** 2021-03-06

**Authors:** Kai Kang, Yue Sun, Dan Pan, Bing Chang, Li-Xuan Sang

**Affiliations:** ^1^Department of Gastroenterology, First Affiliated Hospital, China Medical University, Shenyang, 110001 Liaoning Province, China; ^2^Department of Geriatrics, First Affiliated Hospital, China Medical University, Shenyang, 110001 Liaoning Province, China

## Abstract

Vitamin B (nicotinamide (NAM)), one of the most important nutritional components for humans, exerts anti-inflammatory activity. This study was aimed at investigating the effect of NAM on the gut microbiota and short-chain fatty acids (SCFAs) in mice with chronic colitis. Colitis was induced in C57BL/6 male mice by administration of 1.5% dextran sulfate sodium (DSS), and the mice were intraperitoneally injected with normal saline (NS) or NAM. NAM treatment ameliorated weight loss and changes in colon length, disease activity index (DAI) score, and histologic scores. Moreover, enzyme-linked immunosorbent assay (ELISA) analysis of LPL cells revealed that the level of interleukin- (IL-) 6, IL-12p70, IL-1*β*, tumor necrosis factor- (TNF-) *α*, interferon- (IFN-) *γ*, IL-21, and IL-17A was increased, while IL-10 was reduced, in the chronic colitis group compared to the control group, but the levels of all these factors were restored after NAM treatment. Then, 16S rRNA sequencing of the large intestinal content was performed, and analysis of alpha diversity and beta diversity showed that the richness of the gut microbiota was decreased in the DSS group compared to the control group and restored after NAM treatment. In addition, NAM modulated specific bacteria, including Odoribacter, Flexispira, and Bifidobacterium, in the NAM+chronic colitis group. Phylogenetic Investigation of Communities by Reconstruction of Unobserved States (PICRUSt) analysis indicated that NAM treatment restored disruptions in the functions of the gut microbiota (replication and repair, cell motility) in mice with DSS-induced colitis. Furthermore, NAM also restored the reduction in valeric acid in mice with DSS-induced chronic colitis. Our results suggest that NAM treatment could alleviate DSS-induced chronic colitis in mice by inhibiting inflammation and regulating the composition and function of gut microbiota.

## 1. Introduction

Inflammatory bowel disease (IBD), a chronic immune inflammatory response in the gastrointestinal tract, is classified as 2 major forms, Crohn's disease (CD) and ulcerative colitis (UC) [1, 2]. An increase in the frequency of IBD (from 68.6 cases per 100,000 people in 1990 to 89.6 cases per 100,000 people in 2017) in developing countries has become a significant health problem over the last three decades [3]. Usually, IBD causes symptoms such as fatigue, diarrhea, and abdominal pain [4]. The pathogenesis of IBD is still not fully understood but appears to be influenced by interplay between genetic factors, environmental factors, immunological factors, and disruption of the intestinal microbiota composition [5]. Current treatment options for IBD are mainly based on conventional methods such as treatment with 5-aminosalicylic acid, corticosteroids, and immunosuppressive drugs; however, these therapies generally cause significant side effects and a sizable subset of patient relapse [6, 7]. Therefore, finding effective treatments for IBD is becoming increasingly important.

The intestine is the largest digestive organ in the human body and plays a central role in human defense [8]. CD4+ T cells, which are an important cell population in the colonic lamina propria and epithelium, are vital for the maintenance of intestinal immune homeostasis [9]. CD4+ T cells are divided into subgroups: T helper (Th) 1, Th2, Th17, and regulatory T (Treg) cells. During the inflammatory process, Th1 cells can generate tumor necrosis factor- (TNF-) *α* and interferon- (IFN-) *γ* [10]. Th2 cells produce interleukin- (IL-) 10, which is critical for protection against pathogens [11]. Th17 cells are also involved in the pathogenesis of IBD through the secretion of IL-17A, IL-17F, and IL-22 [12]. Additionally, Treg cells have also been reported to inhibit inflammatory reactions and relieve the development of IBD by secreting immunosuppressive factors such as IL-10 [13].

Many studies have reported that the gut microbiota affects normal physiological and biochemical functions and is associated with a variety of diseases, such as liver disease, cancer, and especially IBD [3, 14, 15]. In a study on IBD, the composition and function of the gut microbiota of patients with IBD were found to be modified, which may result in the impairment of gut physiological function [16]. A decrease in beneficial bacteria, such as Bifidobacterium, was found in patients with IBD [17–19]. Furrie et al. revealed that short-term synbiotic treatment (*Bifidobacterium longum*/Synergy 1) of patients with active UC could improve the clinical appearance of chronic inflammation [20]. *Lactobacillus GG* has also been shown to be effective and safe for maintaining remission in patients with UC [21]. Generally, modulation of the gut microbiota is expected to become an important treatment method for IBD.

Vitamin B, a water-soluble vitamin synthesized by plants, yeasts, and bacteria, has antioxidant properties [22]. Recent studies have reported that vitamin B can maintain the stability of gut microbial communities and plays a critical role in gut diseases [23]. Vitamin B12 deficiency was shown to disturb the gut microbiota in amyloid-*β*-infused rats [24]. A high prevalence of vitamin B12 insufficiency has also been found in patients with CD [25]. The intake of vitamin B6 exhibits a significant inverse association with the severity of IBS symptoms [26]. However, the anti-inflammatory effects of vitamin B3 (nicotinamide (NAM)) in IBD have rarely been reported.

**Figure 1:**
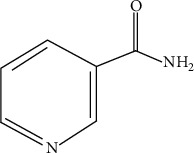
Chemical structure of NAM.

NAM (Figure 1) can be obtained from the diet or through nicotinic acid synthesis [27]. NAM plays an important role in maintaining normal cell function; it was previously shown to effectively prevent cell damage [28]. Additionally, NAM has broad potential application in the treatment of heart-blood vessel disease, respiratory disease, and type 1 diabetes [29]. Smolenska et al. revealed that NAM could be a biomarker of rheumatoid arthritis activity [30]. Kröger et al. also found that NAM could enhance the effect of methotrexate on collagen II-induced arthritis [31]. Furthermore, NAM was reported to protect against colitis by ameliorating pathological angiogenesis and inflammatory changes in a GPR109A-dependent manner [32]. Another study demonstrated that NAM treatment could ameliorate the course of experimental colitis mediated by enhanced neutrophil-specific antibacterial clearance [33]. However, there has been no research on the change of the gut microbiota and its metabolites (short-chain fatty acids (SCFAs)) in the process by which NAM ameliorates IBD.

In the present study, we investigated how dextran sulfate sodium- (DSS-) induced colitis and SCFA disturbances could be reversed by NAM. We analyzed inflammatory mediators, the gut microbiota, and the SCFA composition to evaluate the effect of NAM intervention in colitis. Our results provide evidence for the application of NAM in nutrition therapy for colitis.

## 2. Materials and Methods

### 2.1. Animals

The male C57BL/6 mice utilized in this study were six to eight weeks old and weighed 18–22 g; the mice were purchased from the Changsheng Laboratory Animal Technology Co., Ltd. (Liaoning, China) and group-housed in specific pathogen-free facilities (temperature 23 ± 2°C, humidity 55 ± 5%, and a 12 h light/12 h dark-light regimen). The mice were allowed to adapt to the environment for 1 week before the experiments. For this study, all procedures were performed in strict accordance with National Institutes of Health guidelines and had been approved by the Animal Research Committee of China Medical University (No. 2019068).

### 2.2. Experimental Design

The experimental mice were randomly divided into four groups: A, the control group (*n* = 15); B, the chronic colitis group (*n* = 15); C, the NAM group (*n* = 15); and D, the NAM+chronic colitis group (*n* = 15). The control group received a normal diet, aseptic water, and a once-daily intraperitoneal injection of normal saline (NS) with an equal volume of NAM for 30 d. The chronic colitis group was fed a normal diet and 1.5% DSS and received a once-daily intraperitoneal injection of NS with an equal volume of NAM for 30 d. The NAM group was fed a normal diet and aseptic water and received a once-daily intraperitoneal injection of 250 mg/kg NAM for 30 d. The NAM+chronic colitis group was fed a normal diet with 1.5% DSS and received a once-daily intraperitoneal injection of 250 mg/kg NAM for 30 d. We measured body weight every day and weighed each mouse at the same time daily. We also calculated the survival rate of mice between the chronic colitis group and the NAM+chronic colitis group.

### 2.3. Induction of Chronic Colitis with DSS

**Figure 2:**
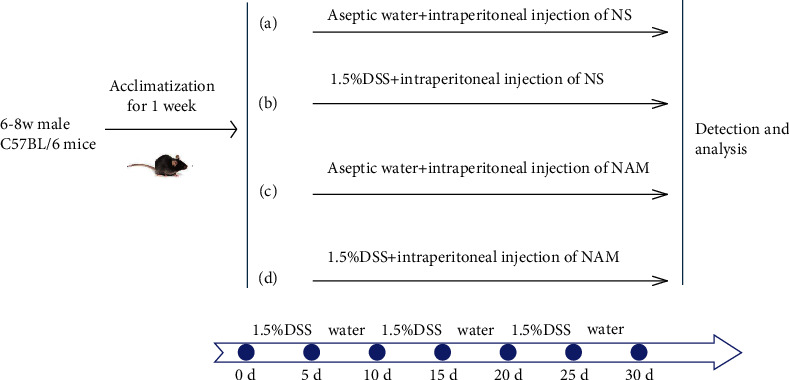
Flow chart of the experiment: (a) control group; (b) chronic colitis group; (c) NAM group; (d) NAM+chronic colitis group.

Colitis was induced in the mice by the administration of 1.5% DSS (molecular mass 36-50 kDa; MP Biomedicals, Solon, OH, United States) on days 0-5, 11-15, and 21-25 d and aseptic water on days 6-10, 16-20, and 26-30 (Figure 2). NAM (C_6_H_5_NO_2_) was purchased from Sigma-Aldrich Corp. (St. Louis, MO, United States).

### 2.4. Sample Collection

On the 30th day, the mice were anesthetized by intraperitoneal injection of 1% sodium pentobarbital (40 mg/kg) and subsequently sacrificed by cervical dislocation. Large intestinal tissues were fixed in a 4% neutral buffered paraformaldehyde solution. The contents of the large intestine were collected and stored at -80°C for further analysis.

### 2.5. Disease Activity Indexing and Histopathology

On the 30th day, all mice were sacrificed by cervical dislocation. The disease activity index (DAI) was used to assess disease severity (Table 1) [34]. We used 4% paraformaldehyde to fix large intestinal tissues and then embedded the tissues in paraffin; hematoxylin and eosin staining was used to evaluate colonic histology in 4 *μ*m thick sections, and the indices in Table 2 were used to assess histological scores. The histological scores were assessed independently by two pathologists blinded to the study groups. In cases of disagreement, comments from a third pathologist were used. The none, mild, moderate, or severe inflammation was quantified as to the percentage involvement by the inflammation: none, 0%-33%, 34%-66%, 67%-100%, and depth of inflammation as to the mucosal damage (none, mucous layer, submucosa, muscularis, and serosa).

### 2.6. Cell Preparation, Culture, and Stimulation

After the mice had been euthanized by cervical dislocation, cut open the abdominal wall, find the ileocecal part and cut the connection between the ileocecal part and the small intestine here, separate the connective tissue around the large intestine with tweezers, dissociate the large intestine to the anus, and cut the rectum. The large intestines were immediately removed and rinsed thoroughly with PBS. The large intestine of each mouse was cut into 1-2 mm pieces on a cold plate. The pieces were stirred for 15 min in PBS (containing 3 mmol/L EDTA) twice and 20 min in RPMI 1640 (containing 1 mmol/L EGTA) twice to eliminate the epithelium at 37°C. The remaining pieces were stirred for 90 min in RPMI 1640 (containing 20% fetal bovine serum, 100 U/mL collagenase (C2139) and 5 U/mL DNase I). Lamina propria cells were used to isolate LPL cells (1 × 10^5^/well in 0.2 mL of RPMI 1640 containing 10% fetal bovine serum, 1% penicillin, and 1% streptomycin) by centrifugation at 2500 rpm with a 45%-66.6% discontinuous Percoll gradient for 20 min. In an atmosphere containing 5% CO_2_, 96-well plates coated with anti-CD3 (10 *μ*g/mL) and soluble anti-CD28 (1 *μ*g/mL) mAbsa1 were cultured at 37°C for 48 h [34, 35]. The supernatant was collected, and cytokines were evaluated by enzyme-linked immunosorbent assay (ELISA) after 48 h.

### 2.7. ELISA

Mouse immunoassay kits (R&D Systems Inc., Minneapolis, MN, United States) were used for analysis; the cell culture supernatants were collected and centrifuged at 1000 rpm for 10 min according to the manufacturer's protocol. The concentrations of the cytokines IL-23, IL-12p70, IL-6, TNF-*α*, and IL-1*β* were measured. The concentrations of the cytokines IL-17A, IFN-*γ*, IL-21, and IL-10 in the supernatants of cells that had been stimulated with or without anti-CD28/anti-CD3 mAbs were then measured [34].

### 2.8. High-Throughput 16S Ribosomal RNA Amplicon Sequencing

Feces samples collected from each mouse on the 30th day were sequenced. 16S rRNA high-throughput sequencing was performed by Shanghai Personal Biotechnology Co., Ltd. (Shanghai, China) using the Illumina MiSeq platform with MiSeq Reagent Kit v3. The laboratory procedures and bioinformatic analysis protocol are provided in detail in the Supplementary Materials and Methods.

### 2.9. 16S rRNA Gene Analysis

OTU-level alpha diversity indices (Shannon index, observed_species), which determined richness and evenness, were calculated using the OTU table in QIIME. Weighed UniFrac nonmetric multidimensional scaling (NMDS) was used to visualize beta diversity (microbial phylogenetic similarity). Analysis of similarities (ANOSIM) was used to assess the significance of structural differences in microbial communities among groups by using QIIME plots. Linear discriminant analysis effect size (LEfSe) was used to detect differentially abundant taxa across groups using the default parameters. According to the obtained OTU relative abundance, R software was used to calculate the number of common OTUs in each group, and the proportions of common and unique OTUs in each group were intuitively presented with a Venn diagram. Species accumulation curves were generated to visualize the total number of OTUs corresponding to each sample in the OTU abundance matrix by using the R package.

### 2.10. Functional Prediction of the Gut Microbiota

16S function prediction was performed by PICRUSt. By comparing the existing 16S rRNA gene sequencing data with a microbial reference genome database with known metabolic functions, the metabolic functions of the bacteria could be predicted, and the differences in 16S rRNA gene copy number between different species were considered in the prediction process. The species abundance data from among the original data were corrected to improve the accuracy and reliability of the prediction results.

### 2.11. Mass Spectrometry for SCFA Analysis

Metabolite extraction was carried out as follows: the samples (large intestinal content) were placed into 2 mL EP tubes, extracted with 0.5 mL of dH_2_O, and vortexed for 10 s. The samples were homogenized in a ball mill for 4 min at 45 Hz and then treated with ultrasound for 5 min (incubated in ice water). The samples were centrifuged for 15 min at 12000 rpm at 4°C. The supernatant (0.3 mL) was transferred into fresh 2 mL EP tubes, extracted with 0.5 mL of dH_2_O, and vortexed for 10 s. The samples were homogenized in a ball mill for 4 min at 45 Hz and then treated with ultrasound for 5 min (incubated in ice water). The samples were centrifuged for 15 min at 12000 rpm at 4°C. Transfer the 0.5 mL supernatant into a fresh 2 mL EP tubes; combine the 0.5 mL supernatant with the 0.3 mL supernatant which was transferred into fresh 2 mL EP tubes, for a total of 0.8 mL of supernatant. A total of 0.1 mL of 50% H_2_SO_4_ and 0.5 mL of 2-methylvaleric acid as an internal standard were added. The mixture was centrifuged for 15 min at 12000 rpm at 4°C. The samples were kept at −20°C for 30 min. The supernatant was transferred to a 2 mL glass vial for GC-MS analysis by using an Agilent 7890 gas chromatograph system coupled with an Agilent 7000D mass spectrometer.

GC-MS analysis was performed using an Agilent 7890 gas chromatograph system coupled with an Agilent 7000D mass spectrometer. The system utilized an HP-FFAP capillary column. A 1 *μ*L aliquot of the analyte was injected in split mode (5 : 1). Helium was used as the carrier gas, the front inlet purge flow was 3 mL min−1, and the gas flow rate through the column was 1 mL min^−1^. The initial temperature was kept at 80°C for 1 min, raised to 150°C at a rate of 5°C min^−1^, and then kept for 12 min at 230°C at a rate of 40°C min^−1^. The injection, transfer line, and quad and ion source temperatures were 240°C, 240°C, 150°C, and 230°C, respectively. The energy was -70 eV in electron impact mode. The mass spectrometry data were acquired in full-scan mode with an *m*/*z* range of 33-200 after a solvent delay of 5 min.

### 2.12. Statistical Analyses of the Data

For host parameters, statistical analyses were performed using SPSS version 25.0 (SPSS Inc., Chicago, IL, United States). Graphs were generated using GraphPad Prism 8 (GraphPad Software, La Jolla, CA, United States). The data are expressed as the mean ± SD, and the body weight and DAI score were analyzed by two-way ANOVA; the pathological damage scores were analyzed by *t*-test; the cytokine was analyzed by one-way ANOVA followed by LSD post hoc test, and previously, a normality test had been applied; when the *P* value was less than 0.05, the difference was considered significant.

For 16S rRNA gene sequencing data, analysis of the gut microbiota was performed by R software. Beta diversity (UniFrac-based weighted NMDS) was determined by the ANOSIM test. Significance in the LEfSe analysis was determined by a logarithmic score from linear discriminant analysis (LDA) difference analysis > 2 and *P* value < 0.05.

For SCFAs, a *P* value below 0.05 and fold change < 0.5 or >2 indicated statistical significance.

## 3. Results

### 3.1. NAM Ameliorated the Severity of DSS-Induced Chronic Colitis

**Figure 3:**
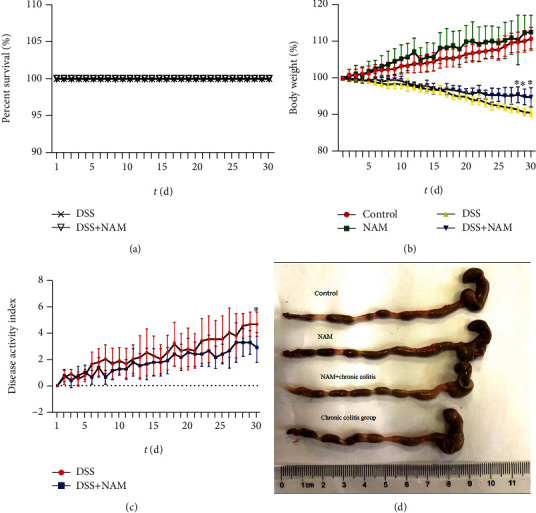
NAM ameliorated DSS-induced chronic colitis: (a) survival rate (*t* (d): time displayed as days); (b) changes in body weight (%); (c) disease activity index (DAI); (d) colon length (the data are presented as the mean ± SD; the body weight and DAI score were analyzed by two-way ANOVA; when the *P* value was less than 0.05, the difference was considered significant. ^∗^*P* < 0.05) (*n* = 8).

**Figure 4:**
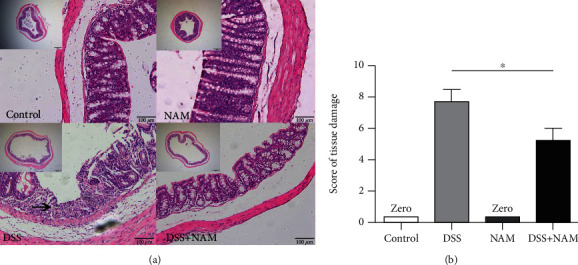
(a) Large intestinal pathology (×40 and ×200): intestinal tissue revealed the infiltration of inflammatory cells and disruption of crypts (the arrow) in the chronic colitis group. (b) Pathological damage scores for the large intestines (the data are presented as the mean ± SD; the pathological damage scores were analyzed by *t*-test; ^∗^*P* < 0.05) (*n* = 8).

The effect of NAM on DSS-induced chronic colitis was evaluated by comparing the survival rate, body weight loss, colon length, DAI score (Figure 3), and large intestinal histology (Figure 4) of mice in the chronic colitis group with those of the NAM+chronic colitis group. There was no difference in survival rate between the chronic colitis group and the NAM+chronic colitis group. The decreases in body weight and colon length were ameliorated in the NAM+chronic colitis group compared with the chronic colitis group. The DAI score of the NAM+chronic colitis group was significantly lower than that of the chronic colitis group.

The effects of DSS-induced chronic colitis were evaluated, and the histological changes are shown in Figure 4(a). The control group and NAM group displayed normal pathological morphologies without colon inflammation. Histological comparison of the intestinal tissue revealed the infiltration of inflammatory cells and disruption of crypts in the chronic colitis group. The pathological damage in the chronic colitis group was ameliorated in the NAM+chronic colitis group. The histological scores were used to assess the infiltration of inflammatory cells. Compared with the chronic colitis group, the NAM+chronic colitis group showed a significantly lower inflammatory score (*P* < 0.05).

### 3.2. Cytokine Production by LPL Cells

**Figure 5:**
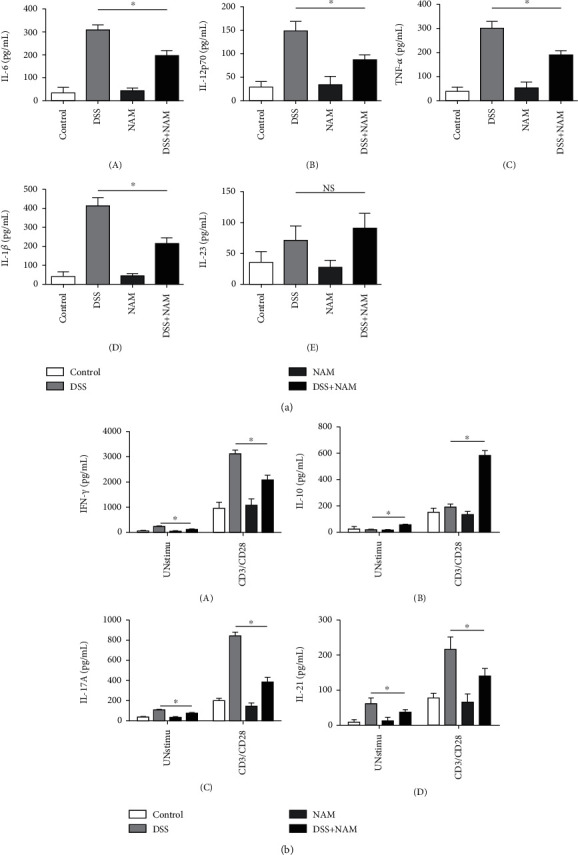
(a) Cytokine production by unstimulated LPL cells was analyzed by ELISA. (b) LPL cells with or without anti-CD3 and anti-CD28 mAb stimulation (the data are presented as the mean ± SD, the cytokine was analyzed by one-way ANOVA followed by LSD post hoc test, and previously, a normality test had been applied; when the *P* value was less than 0.05, the difference was considered significant. ^∗^*P* < 0.05) (*n* = 6).

Changes in the proinflammatory cytokines IL-6, IL-23, IL-12p70, IL-1*β*, and TNF-*α* were analyzed. As shown in Figure 5(a), the levels of IL-6, IL-12p70, IL-1*β*, and TNF-*α* increased after treatment with DSS. However, in the NAM+chronic colitis group, there was a 36% decrease in IL-6 expression (chronic colitis group, mean 309 pg/mL, vs. NAM+chronic colitis group, mean 197 pg/mL), a 48% decrease in IL-1*β* expression (chronic colitis group, mean 414 pg/mL vs. NAM+chronic colitis group, mean 214 pg/mL), a 42% decrease in IL-12p70 expression (chronic colitis group, mean 149 pg/mL vs. NAM+chronic colitis group, mean 86 pg/mL), and a 38% decrease in TNF-*α* expression (chronic colitis group, mean 305 pg/mL vs. NAM+chronic colitis group, mean 188 pg/mL). These results indicate that the production of IL-6, IL-12p70, IL-1*β*, and TNF-*α* was significantly decreased in mice with colitis that had been administered NAM. Thus, NAM had anti-inflammatory activity in mice with DSS-induced colitis by inhibiting the proinflammatory cytokines IL-6, IL-12p70, IL-1*β*, and TNF-*α*.

The concentrations of cytokines that were assayed in the culture supernatants of LPL cells stimulated with anti-CD3 and anti-CD28 mAbs for 48 h are shown in Figure 5(b). The concentrations of IFN-*γ*, IL-21, and IL-17A were significantly lower, while the concentration of IL-10 was significantly higher, in the NAM+chronic colitis group than in the chronic colitis group.

### 3.3. Differences in Microbial Richness and Evenness between Groups

**Figure 6:**
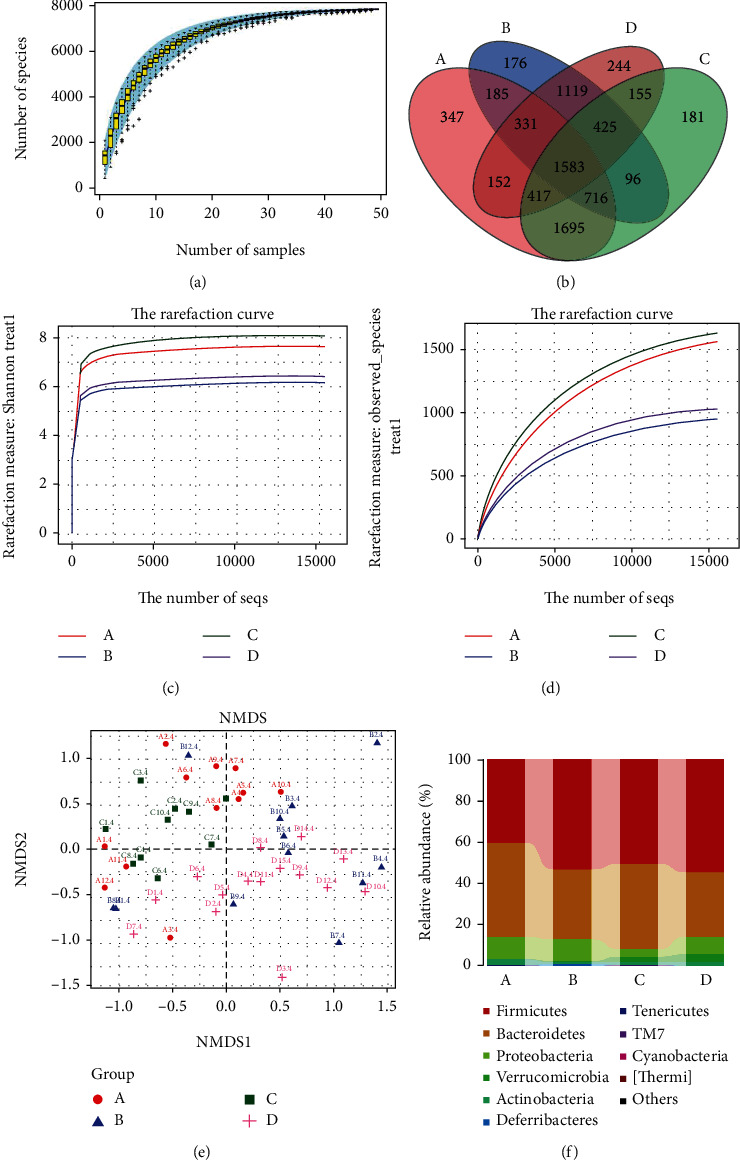
NAM intervention counteracted DSS-induced shifts in the gut microbiota. (a) Species accumulation curves: the species accumulation boxplots reached stable values, indicating that the sequencing covered most phylotypes. (b) Venn diagram of the overlap of operational taxonomic units and differences in operational taxonomic units among groups: 1914 of all OTUs accounting for total richness were universal and found in all samples in the control, chronic colitis, and NAM+chronic colitis groups; 399 OTUs in the NAM+chronic colitis group were not found in the control or chronic colitis group. (c) The alpha diversity index observed_species. (d) The alpha diversity index Shannon index; the alpha diversity index indicated that bacterial species richness was decreased in the chronic colitis group but restored by NAM treatment. (e) The plots shown were generated using weighted UniFrac NMDS. (f) The relative abundance of bacteria at the phylum level in the four groups. (A: control group, *n* = 12; B: chronic colitis group, *n* = 12; C:NAM group, *n* = 10; D: NAM+chronic colitis group, *n* = 15).

The gut microbiota plays an important role in the process of IBD. Microbiota dysbiosis is involved in IBD in patients [36]. To validate the effects of NAM on the gut microbiota, in this study, the v3-v4 region of the 16S rRNA genes from samples (the large intestine content) was amplified by PCR and sequenced. A total of 1861459 sequences were obtained from the 49 samples, with an average of 40333 sequences per control sample, 39933 sequences per chronic colitis sample, and 35727 sequences per NAM+chronic colitis sample. Each sequence was 400-430 base pairs in length (Figure S1). The rarefaction curve reached stability, suggesting that the depth of sequencing covered rare new phylotypes and most diversity (Figure 6(a)).

As shown in Figure 6(b), 1914 of all OTUs accounting for total richness were universal and found in all samples in the control, chronic colitis, and NAM+chronic colitis groups; Venn diagram analysis indicated that some OTUs were common to the 3 groups, and 399 OTUs in the NAM+chronic colitis group were distinct not found in the control or chronic colitis group. DSS significantly affected alpha diversity, leading to a decrease in the Shannon index and observed_species. After treatment with NAM, the Shannon index and observed_species values by DSS were restored to the levels in the control group. Meanwhile, as shown in Figures 6(c) and 6(d), the number of observed OTUs was significantly higher in the control group than in the chronic colitis group. Additionally, the diversity of the NAM+chronic colitis and NAM groups was higher than that of the chronic colitis group. Generally, we found that the decrease in biodiversity in the chronic colitis group was restored by NAM treatment.

### 3.4. Differences in Microbial Community Structure between Groups

To analyze the dissimilarities in gut microbiota community structure among the groups, beta diversity (UniFrac-based weighted NMDS) analysis was performed (Figure 6(e)); NMDS suggested that the structure of the gut microbiota in the chronic colitis group was different from that in the control group, and changes in the gut microbiota structure were observed in the NAM+chronic colitis group (Figure 6(e)). The ANOSIM test showed that the observed cluster patterns were significant (*R* = 0.5581, *P* = 0.001). The microbial structure of the chronic colitis group was significantly different (*R* = 0.5581, *P* = 0.001) from that of the control and NAM+chronic colitis groups. This finding suggests that DSS-induced colitis disrupted the microbial composition and that NAM could shift disruption of the gut microbiota composition caused by DSS.

### 3.5. NAM Intervention Counteracted DSS-Induced Shifts in the Gut Microbiota

The composition of the gut microbiota was influenced by DSS-induced chronic colitis. The top 10 most abundant bacteria at the phylum level are shown in Figure 6(f). The main microbiota at the phylum level included Firmicutes, Bacteroidetes, Proteobacteria, Verrucomicrobia, and Actinobacteria.

**Figure 7:**
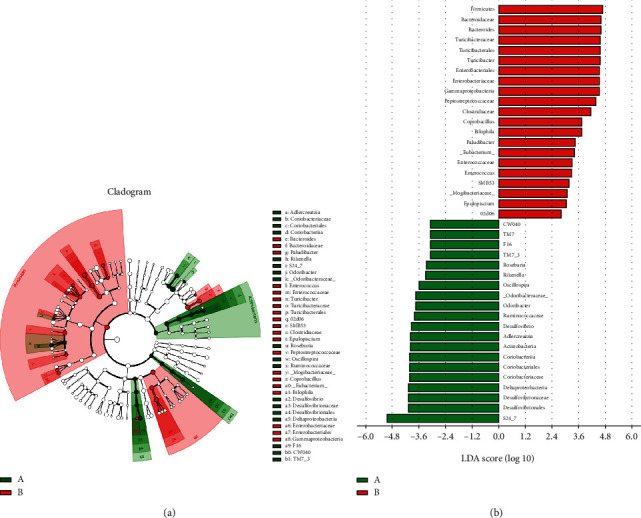
Taxonomic difference in the gut microbiota between the chronic colitis group and control group: (a) a LEFSE cladogram representing different taxa in a tree-like structure; (b) taxa in the control (green) group and chronic colitis (red) group were analyzed by linear discriminant analysis (LDA) effect size. Significantly differentially abundant taxa are listed in the bar plot based on effect size (LDA score (log 10) > 2) and *P* value < 0.05.

To clarify the microbes that were significantly altered in abundance after DSS intervention and NAM treatment, we used the LEfSe method to compare taxa between the DSS and control groups. The structure and dominant bacteria are shown in Figure 7. The analysis revealed that the DSS group was mainly composed of the phylum Firmicutes, family Bacteroidaceae, genus Bacteroides, family Turicibacteraceae, order Turicibacterales, genus Turicibacter, order Enterobacteriales, and family Enterobacteriaceae. The DSS group was mainly composed of the family S24_7, order Desulfovibrionales, and family Desulfovibrionaceae.

**Figure 8:**
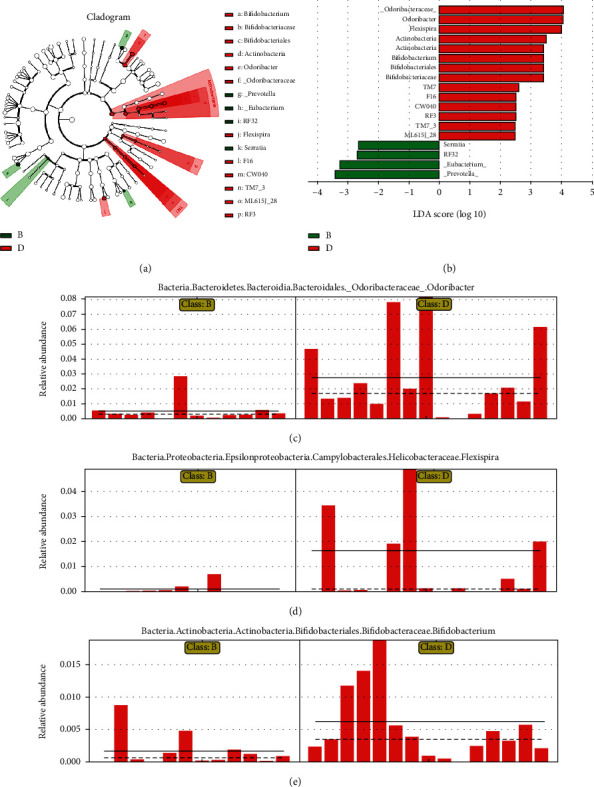
Taxonomic difference in the gut microbiota between the chronic colitis group and NAM+chronic colitis group. (a) A LEFSE cladogram representing different taxa in a tree-like structure. (b) Taxa in the chronic colitis (green) group and NAM+chronic colitis (red) group were analyzed by linear discriminant analysis (LDA) effect size. Significantly differentially abundant taxa are listed in the bar plot based on effect size (LDA score (log 10) > 2) and *P* value < 0.05. (c–e) Significance analysis of differential gut microbial communities in the large intestinal contents of mice in the chronic colitis group and NAM+chronic colitis group. The cladogram and results of LDA are shown. Relative abundances of Odoribacter, Flexispira, and Bifidobacterium in the chronic colitis group and NAM+chronic colitis group are shown. The average and median values for the relative abundance of the taxon in each group are shown by solid lines and dashed lines, respectively, to directly reflect differences between groups. (B: chronic colitis group; D: NAM+chronic colitis group).

In addition, to explore the effects of NAM treatment on the microbiota, taxonomic differences between the chronic colitis group and NAM+chronic colitis group were evaluated. The taxonomic differences between the two groups are shown in Figure 8. The NAM+chronic colitis group was mainly composed of the family Odoribacteraceae, genus Odoribacter, Flexispira, phylum Actinobacteria, class Actinobacteria and genus Bifidobacterium. The chronic colitis group was mainly composed of the genera Prevotella and Eubacterium.

Moreover, we assessed the effect of NAM on the gut microbiome. We used LEfSe to determine microbial differences between the control group and the NAM group (Figure S2). The differentially abundant microbes in the control group included Betaproteobacteria and Burkholderiales. In contrast, the NAM group did not contain microbes with a significantly different abundance. Unlike the significant difference in gut microflora between the control group and chronic colitis group, there were only a few differences in the gut microflora between the control group and NAM group.

### 3.6. Prediction of the Functions of Gut Microbiota

**Figure 9:**
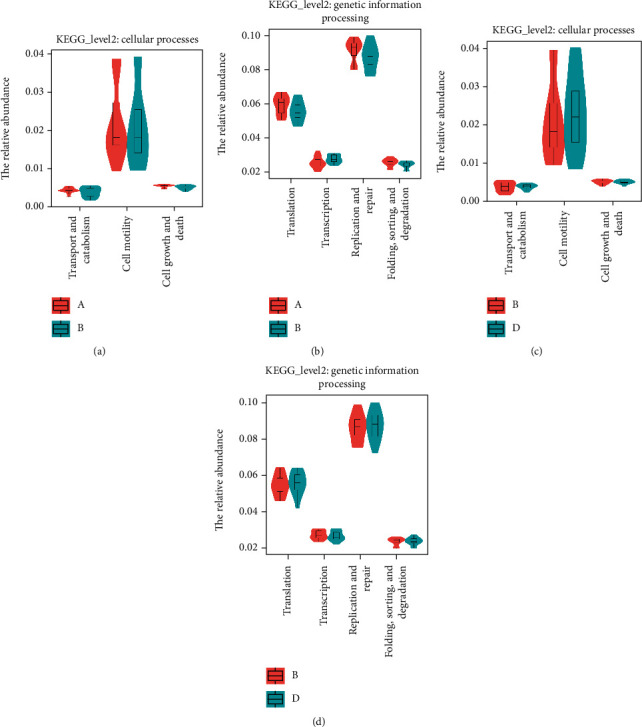
PICRUSt analysis of KEGG pathways. Functional predictions of the gut microbiota in the different groups: (a, b) functional differences in cellular processes and genetic information processing between the control and chronic colitis groups; (c, d) Functional differences in cellular processes and genetic information processing between the chronic colitis and NAM+chronic colitis groups.

To further investigate the shifts in gut bacterial functional profiles related to the changes in gut microbiota in different groups, we analyzed KEGG pathway enrichment in the bacterial populations with PICRUSt. The metabolic pathways included genetic information processing and cellular processes. As indicated in Figure 9, regarding the microbial functions related to cellular processes, the subset related to cell motility was significantly decreased in the chronic colitis group and restored by treatment with NAM.

For DSS-induced mice, as shown in Figure 8, genetic information processing differed among the groups. The replication and repair pathway was less abundant in the DSS-induced colitis group. Surprisingly, the decrease in abundance of members of this pathway was restored after NAM treatment. These results showed that changes in the gut microbiota composition contributed to changes in genetic information processing and cellular processes, which may be associated with the development of colitis.

### 3.7. NAM Intervention Affected the Gut Microbial Ecosystem at the Metabolic Level

**Figure 10:**
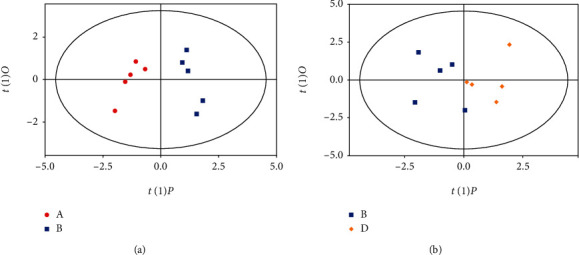
OPLS-DA score plots for the control, chronic colitis, and NAM+chronic colitis groups. Colors indicate the different experimental groups. From the results of OPLS-DA shown as a score chart, we can see very significant distinction among the three groups of samples, and data for all the samples are in the 95% confidence interval.

**Figure 11:**
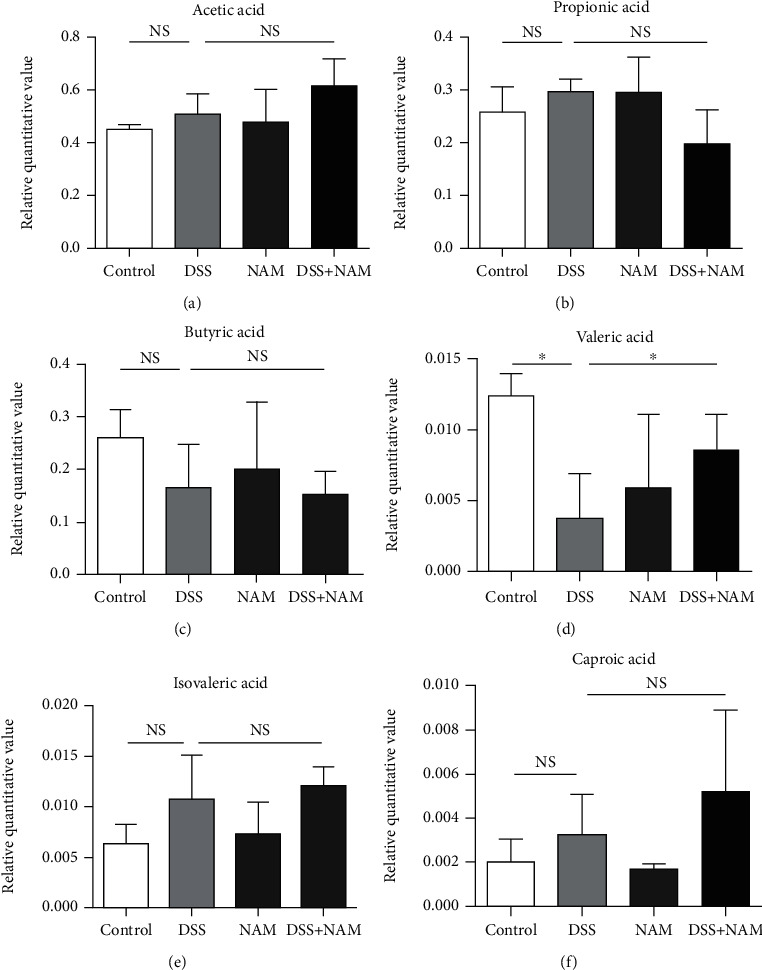
Relative quantitative values of acetic acid, propionic acid, butyric acid, valeric acid, isovaleric acid, and caproic acid concentrations in the large intestinal contents in the control, DSS (chronic colitis), and NAM+chronic colitis groups (*n* = 5 per group). The data are expressed as the mean. Differences were assessed by Student's *t*-test. Significant differences were established at an adjusted *P* value < 0.05 (^∗^*P* < 0.05) with a fold change < 0.5 or >2 (ns: no significant difference).

To assess the effects of DSS and NAM treatment on gut microbes at the metabolic level, SCFAs, which are gut microbiota-dependent metabolites, were quantified. To compare changes in SCFAs in the large intestinal contents of the control, chronic colitis, and NAM+chronic colitis groups, a score scatter plot was generated by orthogonal projections to latent structures discriminant analysis (OPLS-DA) (Figure 10); this plot showed that DSS intervention and NAM treatment caused significant changes in SCFAs. Compared with the valeric acid concentration in the control group, DSS-induced chronic colitis was associated with a significant reduction in valeric acid concentration (control group, relative quantitative value 0.0124 vs. chronic colitis group, relative quantitative value 0.0037), and there was no significant difference in acetic acid, propionic acid, butyric acid, isovaleric acid, or caproic acid concentrations in the control, chronic colitis, and NAM+chronic colitis groups (Figure 11). NAM treatment significantly increased (chronic colitis group, relative quantitative value 0.0037 vs. NAM+chronic colitis group, relative quantitative value 0.0085) and thus restored the levels of valeric acid. Taken together, these results revealed that DSS intervention affected not only the gut microbiota but also the metabolism of SCFAs in gut microbiota.

## 4. Discussion

Although many therapeutic approaches for IBD have been developed, these treatments cause side effects; therefore, new treatment methods are urgently needed. The primary aim of this study was to assess the impact of NAM on DSS-induced colitis in mice, and the second aim was to characterize changes in the gut microbiota and SCFA levels.

Our results demonstrated that 30 d of NAM treatment protected against DSS-induced chronic colitis in mice by attenuating the symptoms of colitis, as shown by measurements of body weight, colon length, and DAI score and by histological assessment of mucosal injury. To further understand the regulatory mechanism of IBD, we analyzed the effect of NAM on inflammatory cytokines at different levels.

Under normal conditions, the inflammatory response in the intestinal mucosa is controlled by a balance between proinflammatory cytokines (such as TNF-*α*, IL-1, IL-6, IL-8, and IL-17) and anti-inflammatory cytokines (such as IL-10). CD4+ Th lymphocytes are responsible for maintaining chronic inflammation in IBD patients [37]. Generally, naive CD4+ Th cells develop into different T helper cell subsets with different cytokine profiles and distinct effector functions. The Th1 response to antigenic stimuli leads to increased levels of various cytokines, such as IL-6 [38]. Th2 cells can synthesize IL-10, an anti-inflammatory cytokine [11, 39]. Different factors (TGF-*β*, IL-6, and IL-23) induce Th17 differentiation, leading to the secretion of IL-17, IL-17F, and IL-22 [12]. Treg cells may indirectly control production of the proinflammatory cytokine TNF-*α* [40, 41]. Previous studies have shown that NAM prompts TNF-*α* production and reduces IL-10 levels in colitis [32]. In another study, NAM was reported to have a potent immunomodulatory effect and may have great potential for the treatment of human inflammatory diseases by reducing the production of IL-1*β*, IL-6, and TNF-*α* [42]. Decreasing levels of IL-6 and IL-1*β* have also been associated with the treatment of colitis [43, 44]. In the present study, significant increases in the production of IL-6, IL-12p70, IL-1*β*, TNF-*α*, IFN-*γ*, IL-21, and IL-17A and decreases in the production of IL-10 were observed in the chronic colitis group compared with the control group, and these changes were restored by NAM treatment. These results demonstrate that NAM ameliorated inflammation in mice with chronic colitis by regulating Th1, Th2, Th17, and Treg cell differentiation by stimulating cytokine secretion. To better understand the immunomodulatory effect of NAM in IBD, further studies at the molecular level are needed.

The human gut is populated by as many as 100 trillion microbial cells [45]. The composition of the gut microbiota codevelops with the host from birth and is subject to complex interplay between the host genome, nutrition, and lifestyle [46]. The principal function of the gut microbiota is to protect the intestine from colonization by exogenous pathogens and potentially harmful indigenous microorganisms, as disturbance of the normal microbial community increases the risk of pathogen infection and inflammatory disease [47]. IBD has been associated with low bacterial richness in the gut [44, 48]. A similar result was observed in the present study, and this low bacterial richness was ameliorated by treatment with NAM. In our study, rarefaction curves became stable, indicating that the sequences had the most diversity and covered few new phylotypes (Figure 6(a)). Meanwhile, the alpha diversity (Figures 6(c) and 6(d)) of the model group was lower than that of the control group. This result suggests that the biodiversity of the model group was lower. Additionally, the alpha diversity of the NAM+chronic colitis group was increased compared with that of the chronic colitis and control groups, suggesting that the alpha diversity of the mice would be restored with NAM treatment.

Gut microbiota dysbiosis has been found in numerous studies [16, 47, 48]. We evaluated the effect of DSS on the gut microbiota by comparing the microbiota of the chronic colitis group and control group at the family and genus levels. In the chronic colitis group, the gut microbiota was mainly composed of the genera Bacteroides (family Bacteroidaceae) and Turicibacter (family Turicibacteraceae) and family Enterobacteriaceae. That of the control group was mainly composed of the families S24_7 and Desulfovibrionaceae. Decreases in biodiversity, such as a decreased proportion of Firmicutes and an increased number of enterobacteria, have been reported in UC and CD patients [49]. Interestingly, an increase in Firmicutes was reported, which is in contrast to the tendency to change observed in our study, which was consistent with a previous study carried out with German IBD patients [50]. Liu et al. reported that after DSS treatment in aged mice, the fraction of harmful bacteria (Turicibacter) was higher than that in young mice [51]. An increase in Bacteroidaceae has also been reported in a DSS-induced colitis mouse model [45, 52]. Another study found that *Ilex kudingcha* could improve DSS-induced colitis by decreasing the abundance of potentially harmful bacteria such as Bacteroides and Turicibacter [53]. The increased level of Bacteroides is also thought to be associated with the development of colon cancer [54]. Wu and coworkers found that chitooligosaccharides could prevent the development of colitis-associated colorectal cancer (CAC) by reducing the abundance of harmful bacteria such as Turicibacter [55]. Furthermore, the presence of Enterobacteriaceae is a common feature of an experimental colitis model [45, 56] and a possible treatment for the altered microbiome of patients with CD [57]. Zhu et al. found that restricting the growth of Enterobacteriaceae decreased intestinal inflammation and reduced the incidence of colonic tumors in CAC, which further confirms the pathogenic role of Enterobacteriaceae [58]. Mice in the colitis progression phase showed reduced levels of Ruminococcaceae S24-7 [59]. Ruminococcaceae S24-7 remain uncultured, but three trophic guilds within S24-7, plant glycan (hemicellulose and pectin), host glycan, and *α*-glucan, were reported to be involved in the degradation of carbohydrates [60]. Therefore, some S24-7 species may have beneficial effects. Taking the above into consideration, the results of our study are consistent with those of a previous report indicating that DSS-induced colitis changes the composition of the gut microbiota mainly by increasing the abundance of pathogenic bacteria (Bacteroides, Turicibacter, and Enterobacteriaceae).

The effect of NAM on the gut microbiota was evaluated by comparing the NAM+chronic colitis and chronic colitis groups. After DSS treatment in aged mice, the fraction of beneficial bacteria (Odoribacter and Alistipes) in the mice was lower [51]. Jiao et al. demonstrated that blueberry polyphenol extract, a potential prebiotic agent, could influence the gut microbiota to positively affect (through increasing Flexispira) high-fat diet-induced obesity in C57BL/6 J mice [61]. The levels of Odoribacter and Flexispira were significantly increased in the NAM+chronic colitis group, which is consistent with a previous study. Another characteristic change in the NAM+chronic colitis group was an increase in the genus Bifidobacterium compared with its abundance in the DSS group. Bifidobacterium is a gram-positive bacterium belonging to the phylum Actinobacteria and represents common inhabitants of the gastrointestinal tract of mammals. This genus can modulate the immune response and protect the intestinal barrier [62]. A previous study reported that *Bifidobacterium longum* LTBL16 has potential as a probiotic [63]. Notably, Bifidobacterium species have been reported to enhance intestinal epithelial barrier function by producing metabolites such as SCFAs [64]. In the present study, the levels of Bifidobacterium were significantly increased in the NAM+chronic colitis group, which agreed with a previous study that revealed the anti-inflammatory effect of Bifidobacterium [65]. Taken together, the results identify that Bifidobacterium may be a novel microbial target for NAM in the treatment of IBD. With the development of sequencing methods, more microbial treatment target of IBD may be found by future studies.

The importance of potential and active functions in the gut microbiota was highlighted by a previous study [66]. Therefore, we used PICRUSt to analyze changes in microbial functions in the gut microbiota communities in the present study. Genetic information processing and cellular processes were altered in the chronic colitis group compared with the control group. Nevertheless, NAM treatment restored the changes in pathway abundance. Regarding genetic information processing, the replication and repair pathway was decreased in the chronic colitis group, which is consistent with previous research showing that genes encoding cell cycle control, replication, recombination, repair, and cell envelope biogenesis were decreased in mice with colitis [67]. However, these dysfunctional changes were abrogated by NAM treatment. This finding suggests that NAM could mend the editing functions of genetic information of gut microbiota. NAM treatment also restored the decrease in cell motility (cellular process) in the DSS group, which is usually found in colitis model mice [68]. Generally, the regulation of genetic information processing and cellular processes suggests that NAM can modulate metabolic pathways in DSS-induced chronic colitis mice and restore maladjusted functions.

While NAM regulates balance of the gut microbiota by stimulating the growth of beneficial bacteria and inhibiting pathogenic bacteria, the gut microbiota also plays an important role in the production of SCFAs [69]. SCFAs, which are microbial metabolites, are an important bridge between the gut microbiota and immune system [70]. SCFAs can suppress colonic inflammation through the activation of Gpr109a (receptor for NAM and butyrate), which induces the secretion of anti-inflammatory cytokines such as IL-10 [71]. Moreover, sodium butyrate was reported to ameliorate colitis in mice with decreased levels of TNF-*α* and IL-6 [72]. Wang et al. revealed that probiotics and fructooligosaccharide can modulate gut microbiota, SCFAs, and serotonin in association with improved autism spectrum disorder symptoms [73]. Thus, gut microbiota-derived metabolites (SCFAs) might play an important role in immunological disorders.

To further confirm the influence of NAM at the metabolic level, we measured the levels of SCFAs in the large intestinal contents of all groups. In the colitis model, DSS significantly decreased the level of valeric acid. The concentration of valeric acid was significantly increased after NAM treatment. Luu et al. reported that valeric acid may suppress autoimmunity by enhancing IL-10 production and suppressing Th17 cells, and valeric acid might be of therapeutic relevance for inflammatory and autoimmune diseases [74]. Another study found that human gut bacteria may inhibit class I histone deacetylase through the production of butyric acid and valeric acid, which has been implicated in a variety of disease pathologies, including colitis and cancer [75]. In children with CD, exclusive enteral nutrition increased the concentrations of anti-inflammatory valeric acids to levels similar to those in healthy age-matched children [76]. Additionally, fecal microbiota transplantation inhibited the growth of *Clostridium difficile* by restoring the levels of valeric acid, further confirming the role of valeric acid [77]. In the present study, NAM intervention significantly restored the levels of valeric acid, indicating that the effect of the treatment is likely associated with the production of valeric acid. These findings suggest that SCFAs have a definite relationship with the development of IBD, but the specific causality still needs further study.

**Figure 12:**
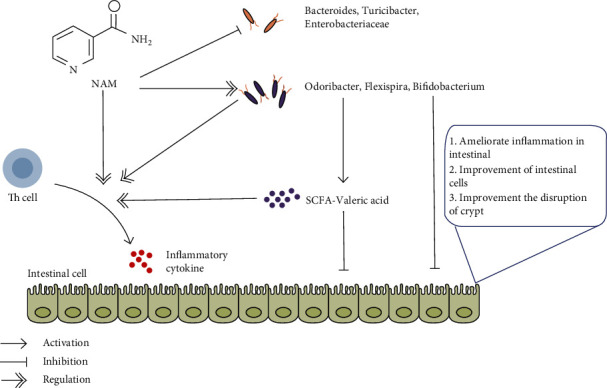
Schematic representation of the proposed anti-IBD effects of NAM on DSS-induced colitis in mice. After the onset of DSS-induced colitis, Th cells are activated, which can increase the production of inflammatory cytokines such as IL-6 and IL-1*β* and decrease production of the anti-inflammatory cytokine IL-10. Moreover, colitis also changes the composition and function of the gut microbiota, which manifests as an increase in harmful bacteria, disrupts the function of the gut microbiota, and disrupts the production of SCFAs. However, the changes of valeric acid can be restored by NAM. On the one hand, NAM can directly protect the gut barrier by regulating the production of inflammatory cytokines, increasing the proportion of the beneficial microbiota, restoring the function of the gut microbiota, and increasing the production of SCFAs. On the other hand, beneficial microbiota and SCFAs can also protect the gut barrier by regulating the production of inflammatory cytokines.

It is well known that altering the nutrient status of a host can reprogram inflammation and metabolic health via the gut microbiota [78]. In the DSS-induced colitis mouse model, Firmicutes and Bifidobacterium were reported to be positively correlated with IL-10 (*P* < 0.05), Enterobacteriaceae was positively correlated with IL-1*β* (*P* < 0.05), and Turicibacter was negatively correlated with IL-10 (*P* < 0.05) [79]. The results of another study showed that Bacteroidaceae was positively correlated with the levels of serum TNF-*α* and mucosal IL-6 (*P* < 0.05) [80]. Wan et al. reported that Bacteroides was positively associated with IL-6 and IFN-*γ*, while the beneficial bacterium Odoribacter was negatively associated with IL-6 (*P* < 0.05) in DSS-induced colitis [53]. Čitar and coworkers found that Bifidobacterium strains could modulate IL-10, IL-6 ,and IL-12 gene expression in THP-1 cells [81]. Additionally, Bifidobacterium has been reported to alleviate DSS-induced colitis by suppressing the IL-17A response and prevent intestinal inflammation through the induction of intestinal IL-10-producing Tr1 cells, further confirming the protective role of Bifidobacterium in colitis [82, 83]. Moreover, the SCFA valeric acid has been reported to suppress autoimmunity by increasing the expression of IL-10 and suppressing Th17 cells [74]. All of these reports are in agreement with our results showing changes in the gut microbiota and inflammatory cytokines (Figure 12).

## 5. Conclusion

In summary, the present study showed that NAM could ameliorate DSS-induced chronic colitis in mice by ameliorating weight loss, changes in colon length, disease activity index (DAI) score, and histologic scores and regulating Th cell differentiation by stimulating cytokine secretion. In addition, our study demonstrated that NAM treatment specifically modulated the bacteria Odoribacter, Flexispira, and Bifidobacterium and restored the disrupted functions of the gut microbiota (replication and repair, cell motility) and proportion of valeric acid, which may be associated with the protective effect of NAM in DSS-induced colitis mice. NAM had an anti-inflammatory effect on DSS-induced chronic colitis in mice and may become a drug for clinical treatment of IBD.

## Figures and Tables

**Table 1:** DAI scores.

Fecal consistency	Fecal occult blood	Decrease in body weight (%)	Integral
Normal	Normal	0	0
		1–5	1

Relaxed	Positive fecal occult blood	>5–10	2
		>10–15	3

Loose stools	Naked eye fecal blood	>15	4

Normal stool: no diarrhea; loose stool: pasty, unformed stools that did not stick to the anus; and liquid stool: diarrhea/liquid stools that did stick to the anus (reproduced from Sang et al. [34] (under the Creative Commons Attribution License/public domain)).

**Table 2:** Histology injury scores.

Feature	0	1	2	3	4
Inflammation	None	Mild	Moderate	Severe	—
Mucosal damage	None	Mucous layer	Submucosa	Muscularis and serosa	—
Crypt damage	None	0%-33%	34%-66%	67%-100%	100% epithelium loss
Range of pathological change	None	0%–25%	26%–50%	51%–75%	76%–100%

## Data Availability

Readers can email the corresponding author for access to the data used in the manuscript.

## Supplementary Materials

Supplementary Materials The supplementary information includes supplementary materials and methods and supplementary figures.
