# Supplementary material for: Nicotinamide Ameliorates Dextran Sulfate Sodium-Induced Chronic Colitis in Mice through Its Anti-Inflammatory Properties and Modulates the Gut Microbiota
Source: J Immunol Res. 2021 Mar 6;2021:5084713. doi: 10.1155/2021/5084713 (PMC7959969; doi:10.1155/2021/5084713)
Supplement: Supplementary Materials — The supplementary information includes supplementary materials and methods and supplementary figures. [file 5084713.f1.doc]

**Supplementary information**

The supplementary information includes supplementary materials and methods and supplementary figures.

1. **High-throughput 16S ribosomal RNA gene sequencing**

Microbial genomic DNA was extracted from fecal samples. The quality of DNA was detected by 0.8% agarose gel electrophoresis, and DNA was quantified by ultraviolet spectrophotometry.

The v3-v4 hypervariable region of the 16S rRNA gene from the microbial genomic DNA obtained from large intestine samples was amplified by polymerase chain reaction (PCR) and used for the rest of the study. PCR primers were designed for the v3-v4 hypervariable region of the bacterial 16S rDNA. The forward primer was 338F 5-ACTCCTACGGGAGGCAGCA-3, and the reverse primer was 806R 5-GGACTACHVGGGTWTCTAAT-3. PCR amplification products were detected by 2% agarose gel electrophoresis, and the target fragments were recovered from the gel.

The recovered PCR amplification products were quantified by fluorescence. The fluorescent reagent used was a Quant-iT PicoGreen dsDNA Assay Kit, and the quantitative instrument used was a microplate reader (BioTek, FLx800).

Raw reads were processed using the Quantitative Insights Into Microbial Ecology (QIIME, v1.8.0) pipeline. Low-quality sequences meeting the following criteria were filtered: 1) sequence length < 160 bp; 2) sequences that contained ambiguous bases; 3) Sequences with 5'end primer mismatch base number > 1; 4) Sequences containing continuous same base number > 8. The remaining high-quality sequences were clustered into operational taxonomic units (OTUs) at 97% sequence identity by UCLUST, and the sequence with the highest abundance in each OTU was selected as the representative OTU. An OTU table was further generated to record the abundance of each OTU in each sample. By comparing the OTU representative sequence with the template sequence of the Greengenes database (Release 13.8), the taxonomic information corresponding to each OTU was obtained. OTUs with an abundance less than 0.001% (1/100,000) of the total sequencing data were removed, and the remaining OTUs were used for subsequent analysis.


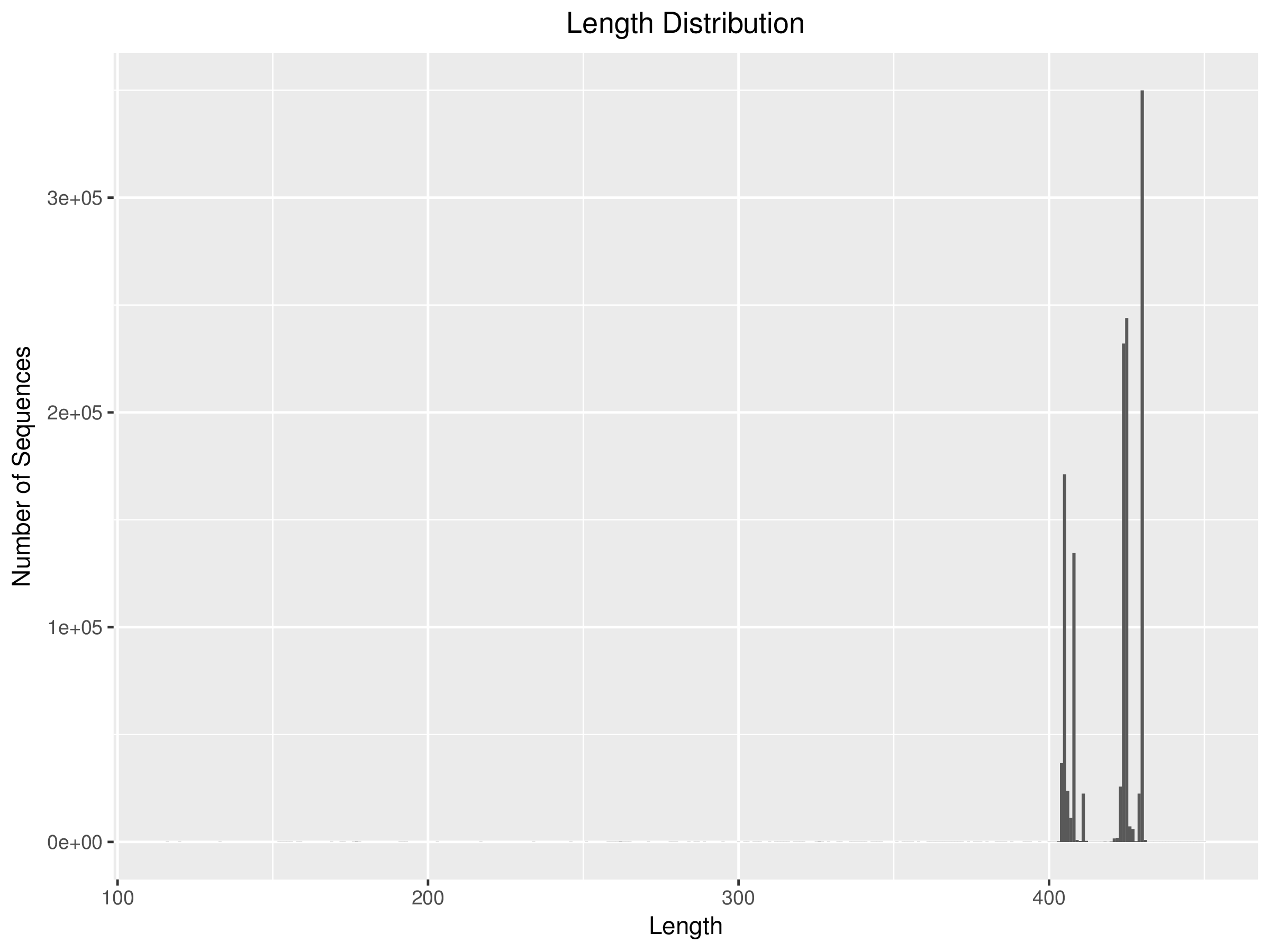


Figure S1**.** Sequence length distribution. The abscissa is the length distribution of the series in all samples, while the ordinate is the total number of series corresponding to each length value.


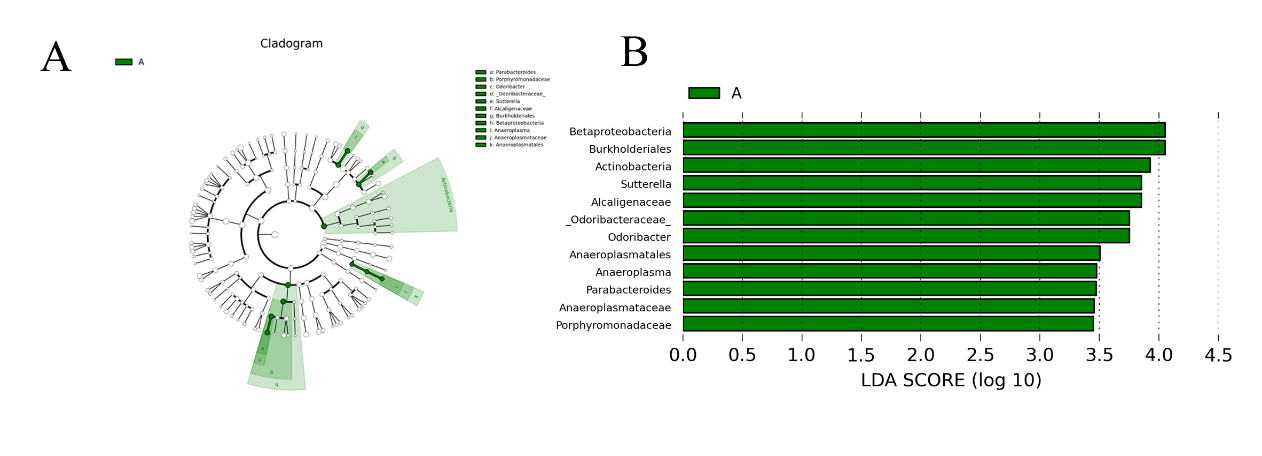


Figure S2**.** Taxonomic differences in gut microbiota between the NAM group and the control group.
